# Hydrophilic High Drug-Loaded 3D Printed Gastroretentive System with Robust Release Kinetics

**DOI:** 10.3390/pharmaceutics15030842

**Published:** 2023-03-04

**Authors:** Gloria Mora-Castaño, Mónica Millán-Jiménez, Isidoro Caraballo

**Affiliations:** Department of Pharmacy and Pharmaceutical Technology, Faculty of Pharmacy, Universidad de Sevilla, 41012 Seville, Spain

**Keywords:** 3D printing, gastroretentive floating tablets, high drug-loaded filaments, FDM, Affinisol™ 15LV, HPMC

## Abstract

Three-dimensional printing (3DP) technology enables an important improvement in the design of new drug delivery systems, such as gastroretentive floating tablets. These systems show a better temporal and spatial control of the drug release and can be customized based on individual therapeutic needs. The aim of this work was to prepare 3DP gastroretentive floating tablets designed to provide a controlled release of the API. Metformin was used as a non-molten model drug and hydroxypropylmethyl cellulose with null or negligible toxicity was the main carrier. High drug loads were assayed. Another objective was to maintain the release kinetics as robust as possible when varying drug doses from one patient to another. Floating tablets using 10–50% *w*/*w* drug-loaded filaments were obtained by Fused Deposition Modelling (FDM) 3DP. The sealing layers of our design allowed successful buoyancy of the systems and sustained drug release for more than 8 h. Moreover, the effect of different variables on the drug release behaviour was studied. It should be highlighted that the robustness of the release kinetics was affected by varying the internal mesh size, and therefore the drug load. This could represent a step forward in the personalization of the treatments, a key advantage of 3DP technology in the pharmaceutical field.

## 1. Introduction

Oral drug delivery has been assumed to have a fundamental role in drug administration, showing advantages such as low therapy cost, easy transportation and storage, and high levels of patient compliance, which makes it adequate for chronic treatments [1].

However, oral administration also presents some physiological limitations due to the heterogeneity of the gastrointestinal tract (GIT) and the variability of the absorption process according to some variables: pH, intestinal microbiota, gastrointestinal transit time, and enzymatic activity. These limitations can lead to an incomplete drug release and to a decrease of the efficacy of the administrated drug [1]. In order to overcome some difficulties that conventional systems could have in the GIT, gastroretentive drug delivery systems (GRDDS) have been designed. GRDDS increase the residence time in the stomach and allow a sustained release of the drug, reducing fluctuations of the therapeutic levels, increasing the bioavailability of the drug and/or allowing its direct administration in the location where a higher therapeutic efficiency could be reached [2,3].

These systems are particularly appropriate for drugs which (i) must have a long-lasting local action in the stomach (ranitidine, amoxicillin, metronidazole), (ii) have a narrow absorption window (metformin, metoprolol, furosemide), (iii) have poor absorption from the lower GIT (atenolol, lafutidine, felodipine), (iv) have low solubility at alkaline pH (ofloxacin, cinnarizine), and (v) are degraded in a basic or neutral environment (verapamil, captopril) [2,4,5,6,7,8].

Different factors such as formulation-related, patient-related, and physiological aspects could affect the quality and performance of the GRDDS in terms of both gastric retention and controlled drug release [9]. Knowledge of these factors is important in the design of GRDDS to guarantee the success of these systems.

Since GRDDS were introduced, various approaches have been followed to achieve gastric retention: expansion or shape changing using different designs with a slim or collapsed configuration that allows swallowing and returns to its original shape in situ [10,11,12,13], muco-adhesion [14,15], high-density systems that sink to the bottom of the stomach, and floating or low-density systems that have lower density than that of the gastric medium (1.004 g/cm^3^) and hence float in the stomach [16,17,18,19,20]. These different approaches can be used by themselves or in combination with each other in the design of GRDDS [21,22].

Comparing all the GRDDS, floating systems seem to provide a promising and practical approach to achieving a long intra-gastric residence time [23,24] due to their low density [7], not affecting the normal motility of the GIT [5], showing a sustained release profile [25,26], and allowing site-specific drug release into the upper GIT for local or systemic effects [27]. All of this means that there are numerous floating gastroretentive systems currently in the clinical trial phase or commercially available [8,9,28]. The commercialized and studied gastroretentive floating (GRF) systems can be categorized into effervescent GRF systems, non-effervescent GRF systems (Hydrodynamically Balanced Systems, Non-Effervescent Tablets, Low-Density Systems) and Raft-Forming Systems [8,9].

GRDDS are usually manufactured by traditional methods such as granulation, compression, etc. However, 3DP technology is recently being proposed as a new technology to produce them [29,30,31,32,33,34].

Three-dimensional printing technology can create solid objects through deposition or polymerization of materials layer by layer, until obtaining the complete object from digital designs [35]. Three-dimensional printing provides an important improvement in the design of floating tablets by producing systems with better time and spatial control of the release of the drug that, at the same time, can be customized based on individual therapeutic needs. In recent years, the most commonly used 3DP technology for the development of GRDDS is FDM, due to its profitability and versatility. This technology allows to obtain different systems with floating capacity: devices in which a conventional tablet containing the drug can be placed inside [30,31,36,37,38,39], as well as systems made of drug-loaded filaments [20,34,40,41,42].

In 3DP floating systems, the type of the polymer, the filling percentage, the drug percentage, and the design of the printed geometrical shape determine the release of the drug and its permanence in the stomach [20,40,42,43]. Currently, the most widely used method for preparing drug-loaded filaments in FDM technology is hot melt extrusion (HME). Both technologies, FDM and HME, have in common the high temperatures needed in the process of obtaining the printed tablet and filaments, respectively [44,45].

Regarding the distribution of the drug in filaments obtained with HME and in the printed tablets, it can be found in the form of solid solution or as a suspension-type system, depending on the temperatures used in their obtention and the physical-chemical characteristics of the components [22,26,46].

Metformin hydrochloride (MET) is a well-known API (active pharmaceutical ingredient) for the treatment of diabetes type II. MET is highly soluble in water but shows a narrow absorption window in the upper GIT with a saturable absorption (BCS Class III). Therefore, MET is a suitable drug for being formulated in a gastroretentive system [47,48]. The thermostability of MET allows its processing by HME and FDM 3DP at a high temperature with no degradation expected [49,50]. Nevertheless, high doses of MET represent an important challenge. Previous studies have shown that the incorporation of a large amount of an API reduces the quality of the filament [49,50]. On the other hand, some cellulose derivatives have been developed in the last years as excipients for HME and FDM 3DP. Cellulose derivatives are well known for their null or negligible toxicity when used as pharmaceutical excipients [51].

Finally, a problem that must be solved to prepare a controlled release drug delivery system aimed for personalized medicine is the fact that the release kinetics of the system must be robust enough to avoid significant changes in the shape of the release profiles when varying the drug dose. This is essential, since each patient would receive the API at different rates depending on their volume of distribution, among other factors.

The aim of this work was to manufacture 3D printed (3DP) tablets designed to (i) keep floating in the gastric medium, (ii) provide a controlled release of the API, (iii) allow a high drug load of MET as a non-molten model drug, using a polymer with null or negligible toxicity based on hydroxypropylmethyl cellulose as the main carrier and a low percentage of auxiliary excipients, and (iv) maintain the release kinetics as robust as possible when the drug doses vary from one patient to another.

## 2. Materials and Methods

### 2.1. Materials

The base polymer in the extruded filaments used to obtain the 3DP tablets was hydroxypropylmethyl cellulose (HPMC) Affinisol™ HME 15 LV (AFF), a hydrophilic, amorphous polymer that was donated by The Dow Chemical Company (Midland, MI, USA). Metformin hydrochloride (MET) was used as the model drug and was donated by Pharmhispania S.A. (Barcelona, Spain). Magnesium stearate (MS) (Fagron Iberica, Terrassa, Spain) was used as a lubricant. Polyethylene glycol 6000 (PEG 6000) (Acofarma, Madrid, Spain) was used as a plasticizer and to facilitate the extrusion process. All percentages are based on *w*/*w*.

### 2.2. Methods

#### 2.2.1. Tablet Design and 3D Printing

The 3D printed cylindrical-shaped systems were obtained using a REGEMAT 3D V1 printer (Regemat 3D S.L., Granada, Spain), using the FDM technique. This technique needs filaments made by melt extrusion, which suffer an extra extrusion by FDM. The extrusion process was performed at 33 rpm and the mixtures were extruded at 150 °C through a 1.75 mm die, using a Noztek Pro Desktop Filament Extruder (Noztek, Shoreham-by-Sea, UK) to obtain the filaments. Different preliminary blends were prepared and tested in our previous work [50] in order to optimize the parameters of the formulation. MS was selected as a lubricant. The enhanced flow speed confirmed that MS facilitates the extrusion process due to its lubricant properties [52,53]. Among the plasticizers tested (PEG 6000 and triethyl citrate) in our previous work, PEG 6000 improved the formulation leading to suitable printable filaments. Metformin and PEG 6000 were sieved and the size fraction 45–180 µm was employed. The weighted average size was approximately 102 µm. The components of the mixtures were mixed using a mortar and pestle. The composition of the drug-loaded filaments used in the current work selected from the previous work [50] is summarized in Table 1. The software REGEMAT 3D DESIGNER v1 was used to design 3DP tablets of a height of 6 mm and a diameter of 12.35 mm. The design included 2 sealing perimeters, 2 bottom solid layers, and 2 top solid layers. Different internal mesh sizes (0.6 × 0.6 mm, 0.8 × 0.8 mm, 1.00 × 1.00 mm) were set to design the 3DP tablets. The printing settings were as follows: nozzle temperature of 200 °C, heated bed temperature of 80 °C, layer thickness of 0.35 mm, nozzle diameter of 0.5 mm, flow speed of 0.75 mm/s, infill and perimeter speed of 4 mm/s, and travelling speed of 15 mm/s.

#### 2.2.2. Characterization of 3DP Tablets

- Thermal analysis

Differential Scanning Calorimetry (DSC) was used to investigate the thermal behaviour of the materials and formulations. The pure substances, physical mixtures, and filaments were studied by using the DSC Q20 167 V24.11 Build 124 (TA Instruments, New Castle, DE, USA). The samples (8–12 mg) were placed in crimped hermetic aluminium pans. They were heated in an atmosphere of 100 mL/min at a ramp rate of 5 °C/min. The starting temperature was 40 °C. The end temperature was 300 °C. Data were analysed at the Functional Characterization Service of the CITIUS in the University of Seville by using a TA Instruments Universal Analysis V4.7A. The thermograms were analysed to detect for thermal events.

TGA was performed to determine the degradation temperature (Td) using a thermogravimetric analyser, Discovery TGA (TA instruments, New Castle, DE, USA). For analysis, 13 ± 4 mg of samples was heated in a nitrogen atmosphere of 100 mL/min at a ramp rate of 5 °C/min, to an end temperature of 300 °C. Data were analysed at the Functional Characterization Service of the CITIUS in the University of Seville by using TRIOS Software (TA instruments, New Castle, DE, USA).

- X-ray powder diffraction

The crystallinity of the powder samples, extruded filaments, and 3DP tablets was studied by X-ray powder diffraction (XRPD) using a Bruker D8 Advance A25 diffractometer (Bruker, Germany) with a copper tube anode, with a 0.02 Ni filter, 0.5° divergence slit scanning range, and LynxEye line detector, at 40 kV and 30 mA. The scanning ranged from 3° to 70° on a 2θ scale and a scanning rate of 9°/min. XRPD was performed by the X-ray Laboratory Service of the CITIUS. Data were analysed using DIFFRAC.SUITE EVA V5.2 software.

- Physical tests of printed systems

The dimensions (height and diameter) and the weights of the 3DP tablets in triplicate were measured using a digital micrometre (Comecta, SA, Barcelona, Spain) and an analytical balance (Sartorius, type LE225D). Equation (1) was used to obtain their densities (*ρ*):

(1) $$\rho =\frac{\mathrm{weight}\;\left(\mathrm{mg}\right)}{\mathrm{diameter}\;\left(\mathrm{mm}\right)/2^{2}\;\times \;\pi \;\times \;\mathrm{height}\;\left(\mathrm{mm}\right)}$$

- Evaluation of surface and inner 3DP tablets

The surface and the inner portions of the samples were analysed using scanning electron microscopy (SEM) and X-ray tomography. SEM was performed at the Microscopy Service of the CITIUS in the University of Seville using a FEI TENEO electronic microscope (FEI Company, Hillsboro, OR, USA) at 5 kV. Samples were previously coated with a 10 nm-thin Pt layer using a Leica EM SCD500 high vacuum sputter coater. X-ray tomography was performed by the X-ray Laboratory Service of the CITIUS in the University of Seville using a Zeiss Xradia 610 Versa (Zeiss, Oberkochen, Germany). The scan was conducted at a peak voltage of 50 kV. Samples were scanned using no filter, an optical magnification of 4×, and a pixel size of 0.88 µm. The image reconstruction was performed using Reconstructor Scout-and-Scan v.16.0, 11, 592 software and exported as a 16-bit tiff file for visualization.

#### 2.2.3. Pharmaceutical Characterization

- Buoyancy study and in vitro drug release

To investigate the buoyancy of the tablets, the float lag time (the time that tablets take to emerge on the surface of a medium) and the total float duration time (the time the tablets remain floating) were observed. In both cases, it was determined visually by the USP Apparatus 2 using 900 mL of pH 1.2 HCl solution maintained at 37 ± 0.5 °C, during 8 h at 50 rpm.

Drug release studies were carried out on the USP apparatus 1 Sotax AT7 Smart, (Sotax, Allschwil, Switzerland) using the same dissolution media as in the buoyancy study. The basket apparatus is more suitable for avoiding the erosion effect of the paddles on the surfaces of the swelling hydrophilic tablets. The study was performed in triplicate for each batch and the solutions were filtered through 0.4 μm microporous membranes. Sink conditions were met throughout the dissolution tests. The percentage of drug released was measured using a UV–vis spectrophotometer Agilent 8453 (Agilent, CA, USA) at a wavelength of 230 nm.

- Drug release kinetics mechanism study

Drug release data (*M_t_*/*M*_∞_ ≤ 0.6) were fitted according to the Zero Order (2), Higuchi [54] (3), Korsmeyer et al. [55] (4), and Peppas and Sahlin [56] (5) equations:

(2) $$\frac{M_{t}}{M_{\infty }}=k_{0}t$$

(3) MtM∞=kt12

(4) $$\frac{M_{t}}{M_{\infty }}=k_{k}t^{n}$$

(5) $$\frac{M_{t}}{M_{\infty }}=k_{d}t^{m}+k_{r}t^{2m}$$

where *M_t_*/*M*_∞_ is the fraction of the drug released at time *t* (drug loading is considered as *M*_∞_). *k* is the Higuchi’s release rate constant. *k_k_* is the Korsmeyer’s kinetic constant, *t* is the release time, *n* is the release exponent that depends on the release mechanism and the shape of the matrix tested [57], *k_d_* and *k_r_* are, respectively, the diffusion and relaxation rate constants, and finally *m*, which is the purely Fickian diffusion exponent for a device of any geometrical shape that exhibits controlled release.

#### 2.2.4. Statistical Analysis

The data are presented as mean ± SD. The statistical analysis was performed by one-way analysis of variance (ANOVA) followed by Scheffe’s post-hoc test using IBM SPSS Statistics Software (Version 26) to analyse the difference in the drug release profiles caused by the studied formulation factors. The significance of the difference has been determined at a 95% confident limit, thus factors showing *p* ≤ 0.05 were considered significant.

## 3. Results

### 3.1. Characterization of 3DP Tablets

#### 3.1.1. Physical Characterization

**Figure 1:**
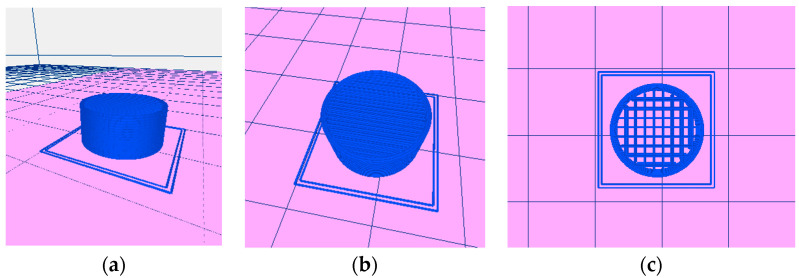
Image of the design of the 3DP tablets: (**a**) perimeters, (**b**) solid top layers, and (**c**) internal mesh.

**Figure 2:**
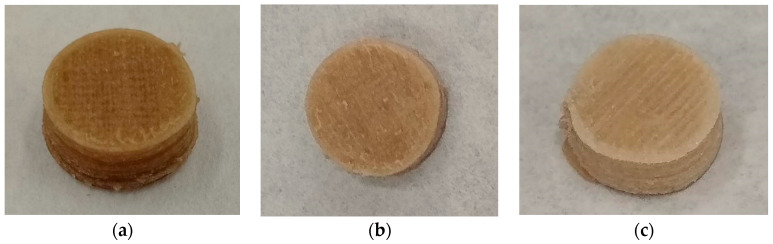
Image of a 3DP tablets made of AFF as main carrier and MET as drug model: (**a**) 30% drug load, (**b**) 40% drug load, and (**c**) 50% drug load.

Three-dimensional printed tablets (Figure 1) were extruded (Figure 2) onto a glass slide, layer by layer by FDM, according to the digitally designed object. The scaffolds were made up of 17 layers with two sealing perimeters in each. Firstly, the FDM extrudes the two bottom solid layers, i.e., in our design, the first two layers are not a network, but a solid layer made up of parallel strands leaving no gaps between them. In the third layer, parallel lines are built. In the fourth layer, the process is repeated, with the difference that the lines are built in a perpendicular direction with respect to the lower ones. Thus, a quadrilateral mesh of different sizes (according to the design) is created. The process continues similarly up to the 15th layer. The FDM extruder then builds two additional solid layers, similar to the first two, to complete the scaffold. All these steps were automatically performed, building the structure without the need for drying steps.

Printed systems were successfully performed using the filaments summarized in Table 1, except filament 50A. In that case, some systems were printed erratically. Thus, this filament was found unsuitable to perform reproducible 3DP tablets in the conditions selected for this study. However, 50B was successfully performed and was included in the drug release studies.

The dimensions and weights of the 3DP tablets of each filament were measured, and their densities were calculated. The results are summarized in Table 2. An optimization was performed using different loads of the drug and auxiliary excipients. Three-dimensional printed tablets containing 5% of each auxiliary excipient were chosen to study the influence of drug loading (10–50%) on drug release profiles. On the other hand, 3DP tablets with representative drug loads were selected to study the influence of the size of the internal mesh and the load of auxiliary excipients.

#### 3.1.2. Thermal Characterization

**Figure 3:**
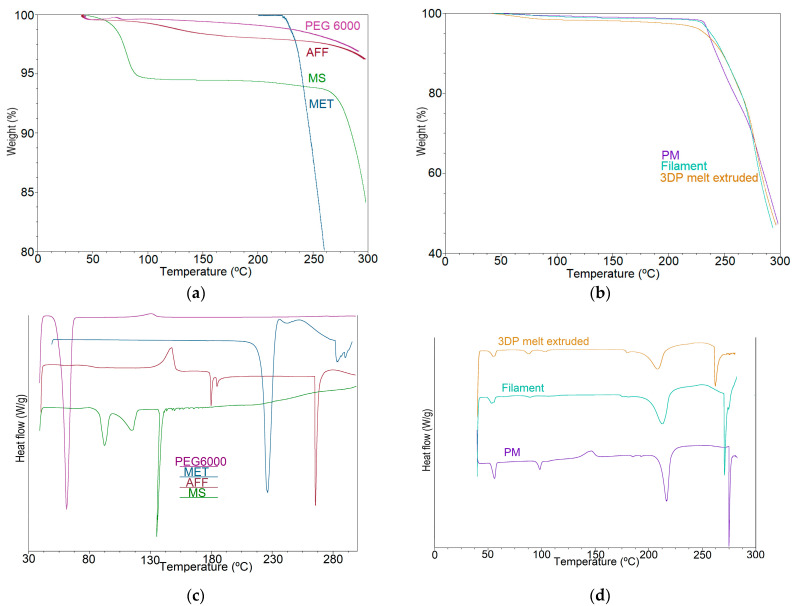
TGA of (**a**) the pure materials and (**b**) the 40B physical mixture, the 40B extruded filament and the 40B 3DP tablet; DSC thermograms of (**c**) the raw materials and (**d**) the 40B physical mixture, the 40B extruded filament and the 40B 3DP tablet.

As shown in Figure 3a, the TGA results show that the drug and excipients were thermally stable at the temperature of the extrusion process (150 °C) and FDM printing (200 °C). All samples (Figure 3a,b) show less than 5% weight loss at 200 °C, indicating their thermostability under FDM conditions.

It should be highlighted that the FDM 3DP temperature (200 °C) was higher than the extrusion process temperature (150 °C), which caused drug-loaded 3DP tablets to turn brownish. Zhao and co-workers also reported this phenomenon, attributing the darker colour of the 3DP AFF tablets to partial HPMC cooking in the curing steps instead of degradation [26]. DSC and TGA analysis results confirm this hypothesis (Figure 3). Figure 3b,d show TGA and DSC of the drug load of 40% and 7.5% of auxiliary excipients (physical mixture (PM), filament, and 3DP tablets), which was chosen as an example.

The DSC results (Figure 3c) showed that crystalline metformin hydrochloride had a sharp endothermic peak at 229 °C, which was a melting peak. However, a slight shift of the peak temperature of MET was observed for the filaments compared to that obtained for pure MET [50]. The melting peak appeared in PM, drug-loaded filaments, and 3DP tablets (Figure 3d). Nevertheless, the peak intensity was PM > drug-loaded filaments > 3DP tablets, from high to low, which could be attributed to a partial amorphization of the drug during the extrusion process and FDM 3DP [26,40]. This results in a wider melting range, or a melting process that starts at lower temperatures.

Furthermore, this effect can also be observed in the AFF peak shift. The 260 °C AFF peak shown in Figure 3c appears at a lower temperature in the thermograms of filament samples, and even lower in those of 3DP tablets (Figure 3d).

#### 3.1.3. X-ray Powder Diffraction (XRPD)

**Figure 4:**
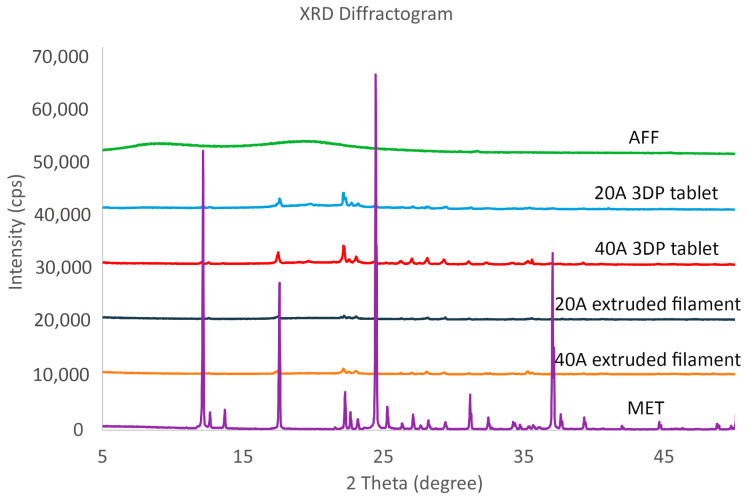
XRPD patterns of MET, AFF, 20A, and 40A extruded filaments, and 20A and 40A 3DP tablets.

XRPD analysis was performed to further determine the dispersion pattern of the drug in the samples (Figure 4). Moreover, the crystallinity percentage of each sample was analysed using a semiquantitative method. The results were: 35.8% (AFF), 51.4% (20A 3DP tablet), 65.5% (40A 3DP tablet), 48.6% (20A filament), 61.7% (40A filament), and 93% (MET). In agreement with the DSC results, the sharp 2θ diffraction peaks of MET at 12, 17, 24, 31, and 37° [58,59] appeared much lower in drug-loaded extruded filaments and 3DP tablets. The peak intensity was higher in MET sample than in extruded filaments and 3DP tablets samples. Moreover, the extruded filaments and 3DP tablets diffractograms are smoother than that of MET. All these tendencies and data suggest that a portion of the drug crystals transformed into amorphous after the extrusion process and FDM 3DP steps, and the drug was dispersed into the polymer matrix [26]. It should be highlighted that the peak intensity is higher in 3DP tablets than in extruded filaments diffractograms.

### 3.2. Pharmaceutical Characterization

#### 3.2.1. Buoyancy of the 3DP Tablets

All the printed systems placed in the dissolution media floated immediately, thus the floating lag time was 0 s. The systems kept floating for more than 8 h.

Considering the different variables in the design and composition of the systems studied in this work (internal mesh size, drug load, auxiliary excipients load), the main factor involved in the buoyancy was the density of the 3DP tablets, in agreement with the previous literature [39,40]. The density of our printed systems ranged from 0.73 to 0.82 g/mL (Table 2). Three-dimensional printing technology allows the design of tablets containing a customized internal mesh. Furthermore, the design of the obtained 3DP tablets, sealed by two perimeters in each layer, two solid bottom layers, and two solid top layers, allows to capture the air inside, making them float from the first moment. Consequently, all the 3DP tablets were successful 3DP floating tablets.

The excipients derived from cellulose (HPMC among them) have the advantage of a low or null toxicity [51]. Other studies obtaining 3DP floating tablets using HPMC as the main carrier have been reported. However, those tablets contained low drug loads (10–30%) and additional carriers besides HPMC [26,42,46]. In the present study, buoyant 3DP tablets containing high drug loading have been successfully obtained for the first time using a cellulose-based carrier, without any additional polymers.

#### 3.2.2. Drug Release Behaviour

- Effect of drug load

**Figure 5:**
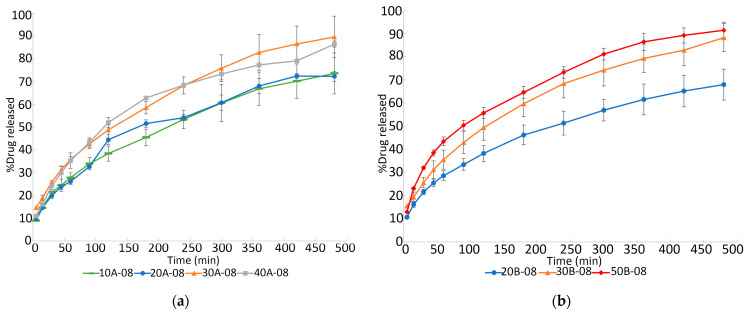
Effect of drug load on the in vitro dissolution of 3D 3DP tablets: (**a**) 5% of each auxiliary excipients; (**b**) 7.5% of each auxiliary excipient.

The influence of drug load on the in vitro dissolution studies is shown in Figure 5. Three-dimensional printed tablets of internal mesh size of 0.8 mm × 0.8 mm were chosen to study the influence of drug load on the drug release profiles. The dissolution profiles of 10% and 20% drug load 3DP floating tablets were significantly different from those of the 30% and 40% drug load systems at all time points, containing 5% of each auxiliary excipient (Figure 5a). Nevertheless, significant differences were not found between 10% and 20% drug load, nor between 30% and 40% drug load. Around 70% of the drug released was achieved after 8 h of the dissolution test in printed systems containing 10% and 20% of the drug. However, around 90% of the drug release was achieved in the same range of time for those which contained 30% and 40% of the drug. Significant differences were also found between 20% and 30% drug-loaded filaments containing 7.5% of each auxiliary excipient (Figure 5b), whereas differences between 30% and 50% drug load were not statistically significant. These data confirm the positive influence of the drug load on the dissolution rate. It is important to realize that the present paragraph is not referring to the weight of the API included in the GRDDS, but to the percentage of the drug loaded in the GRDDS, which in turn corresponds to the percentage of the API included in the filaments. Obviously, the drug load depends on the percentage of the drug in the GRDDS and on the weight of the GRDDS.

**Figure 6:**
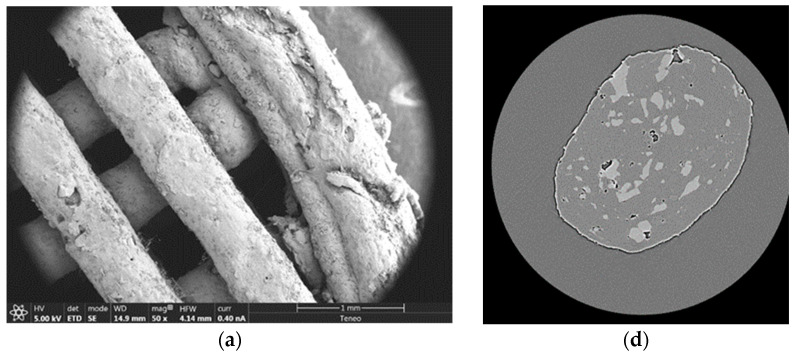

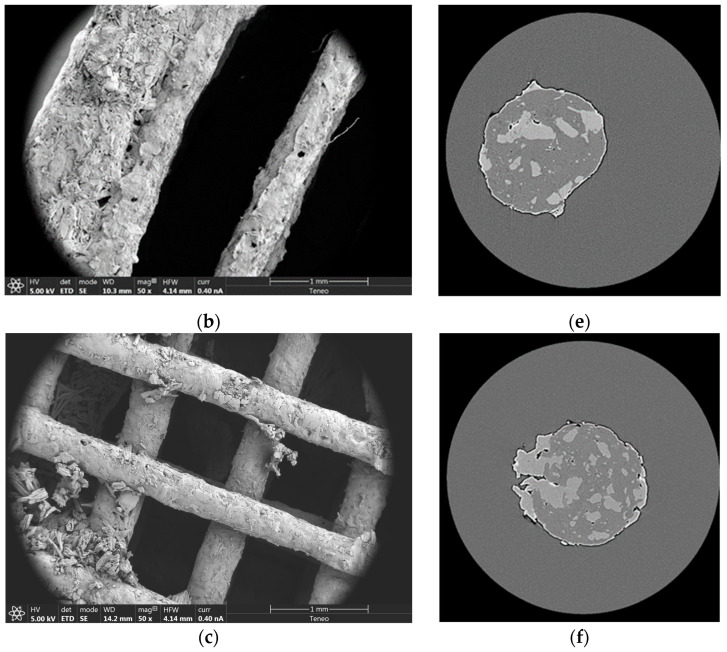
SEM images at 50X of the inner structure of the systems printed with 20% (**a**), 30% (**b**), and 50% (**c**) drug loading, and images made with X-ray tomography of the cross-section of the strands of the same drug percentages (20% (**d**), 30% (**e**), and 50% (**f**)).

As can be seen in Figure 6a–c, metformin crystals appeared on the surface of the 3DP floating tablets containing 30% of the drug. However, this was not the case for the 20% drug-loaded 3DP tablets. Figure 6d,e, and f show an X-ray tomography of the cross section of the strands of the 3DP tablets. The lighter color represents metformin. As can be seen, the higher the drug load, the more metformin is located outside the strands. This can be attributed to the partial drug recrystallization process shown in the XRPD results (Figure 4). Furthermore, it can be seen that this process is more pronounced as the drug load increases (Figure 6). From 30% drug loading onwards, metformin transitioned from the matrix to the surface. This could explain the difference in drug release behavior found between the two data sets (10–20% vs. 30–40% drug loading). There would be more metformin on the surface in the case of 30% and 40%. This drug will quickly come into contact with the dissolution medium, which could explain why the dissolution rate of 3DP tablets with 30–40% drug loading is faster than those with 10–20% metformin loading.

- Effect of auxiliary excipients load

**Figure 7:**
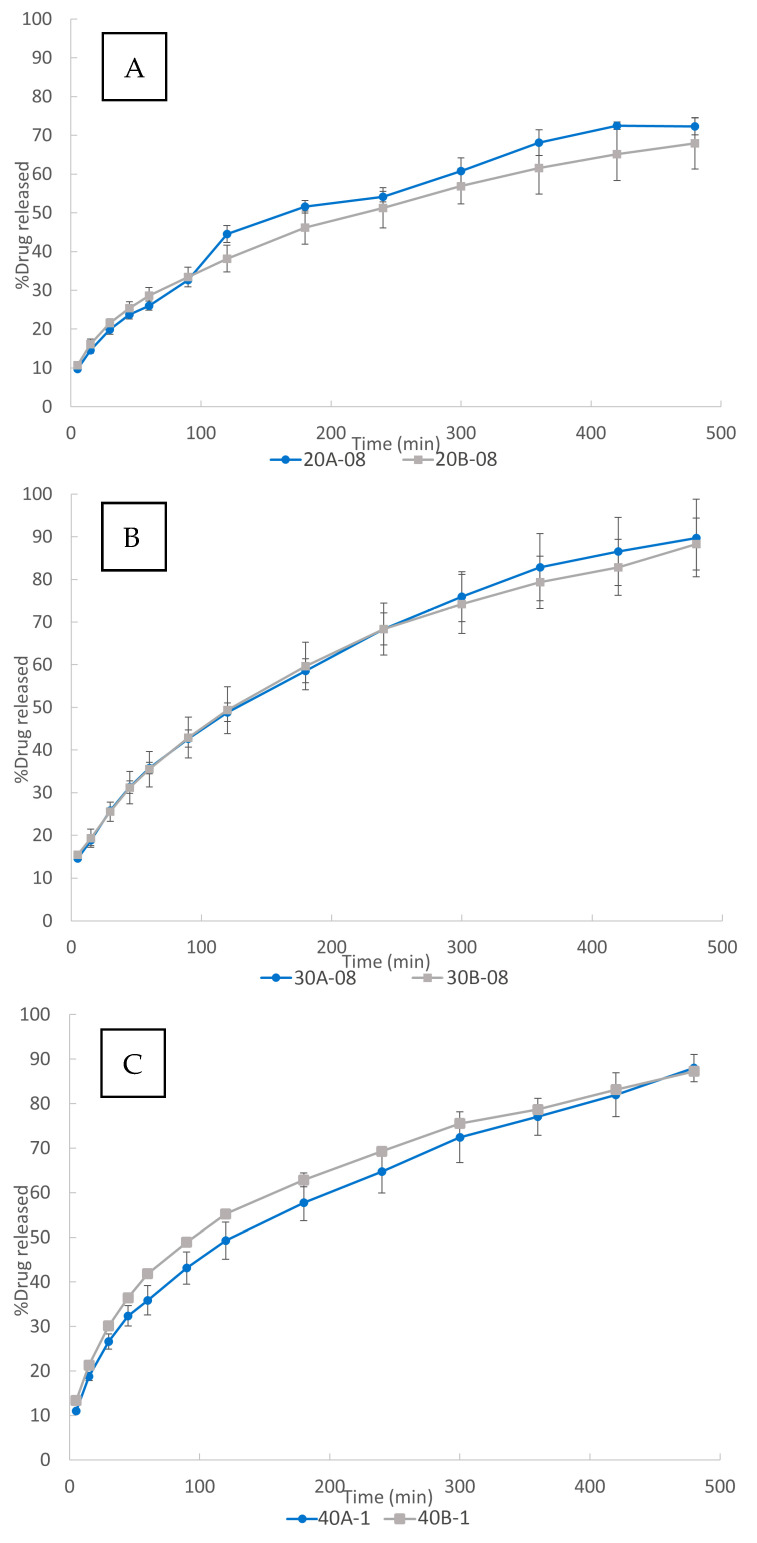
Effect of auxiliary excipients load on the vitro dissolution 3DP tablets: (**A**) 20% drug load, (**B**) 30% drug load, and (**C**) 40% drug load.

The influence of the percentage of auxiliary excipients on the in vitro dissolution studies is shown in Figure 7. As can be observed, significant differences were not found between 3DP tablets containing 5% and 7.5% of auxiliary excipients. This result is in agreement with the previously reported work by Vo and co-workers, who did not find a significant effect of the stearic acid content in the 3DP systems on the drug dissolution rate [60].

In the present work, the absence of a clear effect could be attributed to the opposite nature of the employed auxiliary excipients (MS and PEG 6000). PEG 6000 is hydrophilic and could lead to an increase in the drug dissolution rate. MS is hydrophobic and could lead to a slower drug dissolution rate. Results in Figure 7 showed that the drug dissolution rate was not modified when varying the percentage of the auxiliary excipients. This could be attributed to a compensation of the effects of the employed excipients, since they have opposite natures.

- Effect of internal mesh size

**Figure 8:**
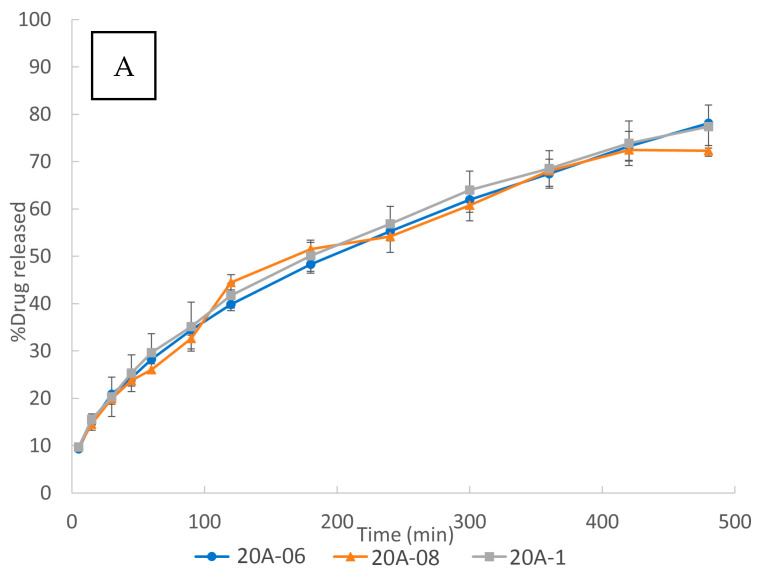

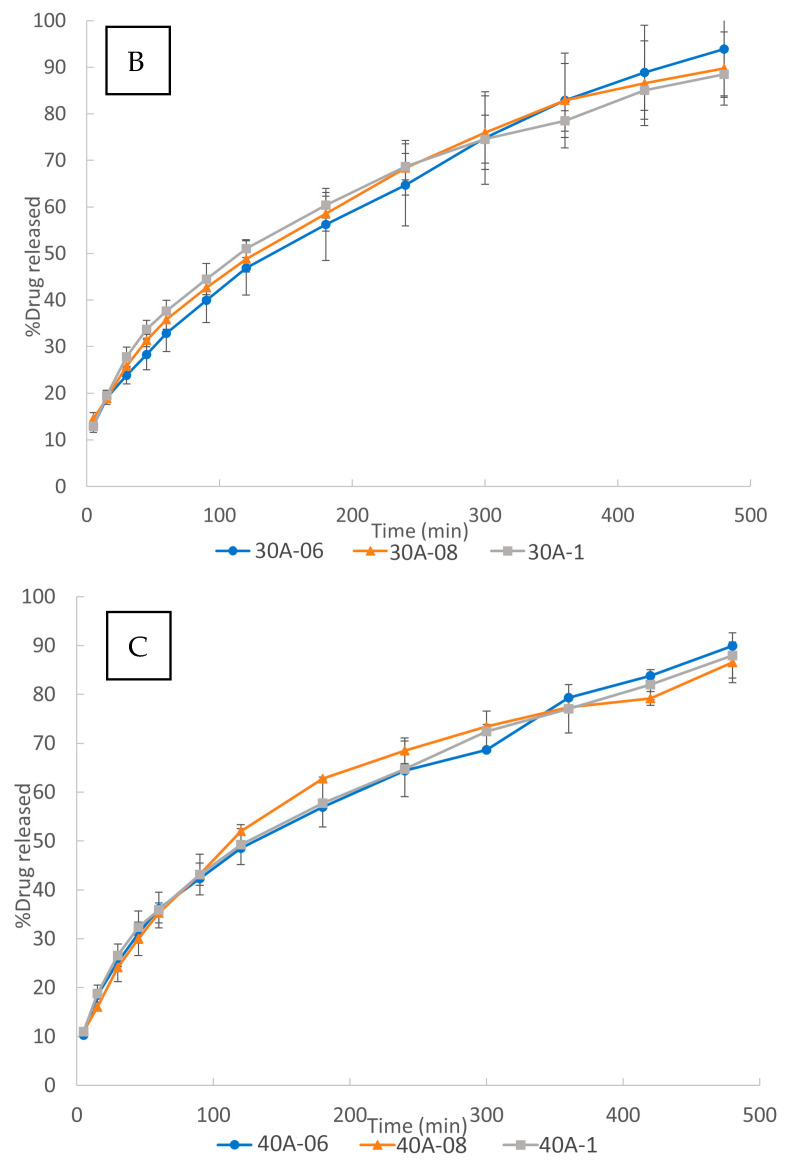
Effect of the internal mesh size of 3DP tablets: (**A**) 20% drug load, (**B**) 30% drug load, and (**C**) 40% drug load.

The influence of internal mesh size on the in vitro dissolution studies is shown in Figure 8. Although most of the authors showed an influence of the internal mesh size on the dissolution rate [32,33,39,42,61] of their 3DP scaffolds, as Figure 8 shows, variation of the internal mesh size did not result in a significant effect on the drug dissolution for the 3DP floating tablets developed in the present work. This phenomenon can be mainly attributed to their design:

The performed 3DP floating tablets are sealed by two perimeters in each layer, two solid bottom layers, and two solid top layers, as described in Section 3.1. The AFF sealing layers form a gelled barrier, being the main factor conditioning the drug release. Thus, the release was mainly controlled by the external structure of the scaffold (two perimeters and the two + two solid layers). This could explain the lack of influence of the internal mesh size of the inner scaffold on the drug release.

A second factor which could help to reduce the hypothetical influence of the mesh size could be a compensation between the water uptake rate and the diffusion rate. On one hand, the water uptake rate is expected to be higher for the smaller internal mesh size due to the capillarity, since the polymer is hydrophilic.

Therefore, the aqueous dissolution medium would penetrate through the matrix faster than into the 3DP floating tablets of a bigger internal mesh size. On the other hand, the smaller internal mesh size could make the drug diffusion from the polymer matrix to the dissolution test medium slower. The compensation between both phenomena could lead to a decrease in the influence of the internal mesh size on the drug dissolution release. In any case, the influence of this second factor, if any, is expected to be lower than the first one.

The fact that the internal mesh size did not influence the relative release rate of the API, i.e., the percentage of API released at any time point, supposes an enormous practical advantage in personalized medicine: based on this characteristic of the designed structures, it is possible to adjust the dose of the treatment just by varying the internal mesh size. By changing the mesh size, the weight of the GRDDS can be tuned according to the needs of the patient. Nevertheless, thanks to the design of the proposed systems, the release kinetics will be maintained. In other words, the drug dose received by each patient would be different, whereas the shape of the release profiles will be the same.

#### 3.2.3. Drug Release Kinetics

Dissolution data were analysed using the different kinetic equations of Section 2.2.3. (Table 3). The good determination coefficients (*r*^2^) of the drug release profiles for the Higuchi’s model reveal a diffusion mechanism for the systems. Korsmeyer n values close to 0.45, and the predominance of *k_d_* values from the Peppas and Sahlin model, confirm the diffusional mechanism for all the studied 3DP floating tablets.

## 4. Conclusions

Drug-loaded filaments composed of Affinisol™ 15LV as a carrier, auxiliary excipients (PEG 6000, magnesium stearate), and metformin hydrochloride were developed and proved reliable for preparing FDM 3DP floating controlled-release tablets. All of the 3DP tablets floated successfully with no floating lag time, thanks to the 3D design performed in this work. The sealing layers allowed keeping the air inside, maintaining the low density of the tablets. This resulted in buoyancy for more than 8 h and a simultaneous diffusion-controlled release of the API. Furthermore, this work showed for the first time that 3DP floating tablets containing up to 40% of the drug were obtained using a cellulose-based polymer as a carrier, without any additional polymers.

On the other hand, the effect of different variables on the drug release behaviour has been studied. It should be highlighted that the robustness of the release kinetics was affected when varying the internal mesh size, and therefore, the internal porosity of the system and its weight. This way, the dose can be safely and easily adjusted for each patient, keeping the release kinetics unaffected. This supposes a step forward in the personalization of treatments, a valuable advantage of 3DP technology in the pharmaceutical field.

## Figures and Tables

**Table 1:** Composition of the drug-loaded filaments. The letter (A/B) refers to the concentration of both auxiliary excipients (MS and PEG 6000), A corresponding to 5% each and B to 7.5% each.

Drug-Loaded Filament	MET (%)	AFF (%)	MS (%)	PEG 6000 (%)
10A	10	80	5	5
20A	20	70	5	5
30A	30	60	5	5
40A	40	50	5	5
50A	50	40	5	5
20B	20	65	7.5	7.5
30B	30	55	7.5	7.5
40B	40	45	7.5	7.5
50B	50	35	7.5	7.5

**Table 2:** Physical characterization of the 3DP tablets. The numbers after the hyphen indicate the mesh size, being 06 = 0.6 mm × 0.6 mm, 08 = 0.8 mm × 0.8 mm, and 1 = 1.0 mm × 1.0 mm.

3DP Tablets	Internal Mesh Size (mm × mm)	Height (mm)	Weight (mg)	Diameter (mm)	Density (g/mL)
20A-06	0.6 × 0.6	6.33 ± 0.34	669.74 ± 18.03	13.00 ± 0.21	0.80 ± 0.03
30A-06	0.6 × 0.6	5.94 ± 0.01	626.29 ± 7.33	13.30 ± 0.14	0.76 ± 0.01
40A-06	0.6 × 0.6	6.21 ± 0.11	683.71 ± 17.71	13.27 ± 0.16	0.80 ± 0.03
10A-08	0.8 × 0.8	6.22 ± 0.06	687.60 ± 5.00	13.25 ± 0.18	0.80 ± 0.02
20A-08	0.8 × 0.8	6.21 ± 0.16	666.80 ± 42.14	12.95 ± 0.07	0.82 ± 0.02
30A-08	0.8 × 0.8	6.10 ± 0.02	644.97 ± 6.24	12.93 ± 0.03	0.80 ± 0.01
40A-08	0.8 × 0.8	6.37 ± 0.13	652.80 ± 5.87	13.01 ± 0.03	0.77 ± 0.02
20B-08	0.8 × 0.8	5.97 ± 0.18	635.56 ± 9.05	13.24 ± 0.17	0.77 ± 0.02
30B-08	0.8 × 0.8	5.59 ± 0.54	617.58 ± 14.22	13.13 ± 0.23	0.82 ± 0.04
50B-08	0.8 × 0.8	5.32 ± 0.08	594.70 ± 24.98	13.54 ± 0.21	0.78 ± 0.06
20A-1	1.0 × 1.0	6.15 ± 0.18	626.64 ± 29.81	13.33 ± 0.40	0.73 ± 0.04
30A-1	1.0 × 1.0	5.77 ± 0.21	620.83 ± 1.74	13.40 ± 0.00	0.76 ± 0.03
40A-1	1.0 × 1.0	6.22 ± 0.14	618.92 ± 25.36	13.19 ± 0.20	0.73 ± 0.01
40B-1	1.0 × 1.0	6.21 ± 0.16	666.80 ± 42.14	12.95 ± 0.07	0.82 ± 0.02

**Table 3:** Drug release kinetic parameters of the 3DP tablets. B, Higuchi kinetic constant; *n*, release exponent; *k_d_*, Peppas diffusion kinetic constant; *k_r_*, Peppas relaxation kinetic constant; *r*^2^, determination coefficient.

3DP Tablets	Zero Order		Higuchi		Korsmeyer		Peppas and Sahlin		
	*k*	*r* ^2^	*b*	*r* ^2^	*n*	*r* ^2^	*k_d_*	*k_r_*	*r* ^2^
20p06A	0.1871	0.9540	0.0348	0.9997	0.4622	0.9989	0.0344	0.0000	0.9997
30p06A	0.2003	0.9568	0.0391	0.9965	0.4481	0.9918	0.0308	0.0005	0.9989
40p06A	0.2529	0.9241	0.0419	0.9959	0.4780	0.9979	0.0534	−0.0007	0.9996
10p08A	0.1760	0.9433	0.0334	0.9983	0.4634	0.9987	0.0331	0.0000	0.9983
20p08A	0.1822	0.9700	0.0336	0.9965	0.4461	0.9939	0.0263	0.0004	0.9988
30p08A	0.2331	0.9647	0.0404	0.9979	0.4572	0.9999	0.0373	0.0002	0.9986
40p08A	0.3496	0.9797	0.0475	0.9949	0.4981	0.9904	0.0332	0.0011	0.9985
20p08B	0.1469	0.9434	0.0305	0.9991	0.4111	0.9992	0.0339	−0.0002	0.9997
30p08B	0.2395	0.9732	0.0407	0.9957	0.4581	0.9990	0.0318	0.0006	0.9981
50p08B	0.3492	0.9138	0.0490	0.9900	0.4604	0.9949	0.0735	−0.0019	1.0000
20p1A	0.1937	0.9521	0.0361	0.9989	0.4622	0.9975	0.0360	0.0000	0.9989
30p1A	0.3179	0.9479	0.0440	0.9979	0.4394	0.9978	0.0505	−0.0005	0.9988
40p1A	0.2531	0.9247	0.0419	0.9961	0.4645	0.9983	0.0532	−0.0007	0.9996
40p1B	0.3503	0.9401	0.0486	0.9970	0.4535	0.9990	0.0602	−0.0009	0.9993

## Data Availability

Data are contained within the article.
